# deML: robust demultiplexing of Illumina sequences using a likelihood-based approach

**DOI:** 10.1093/bioinformatics/btu719

**Published:** 2014-10-30

**Authors:** Gabriel Renaud, Udo Stenzel, Tomislav Maricic, Victor Wiebe, Janet Kelso

**Affiliations:** Department of Evolutionary Genetics, Max Planck Institute for Evolutionary Anthropology, Leipzig, Saxony D-04103, Germany

## Abstract

**Motivation:** Pooling multiple samples increases the efficiency and lowers the cost of DNA sequencing. One approach to multiplexing is to use short DNA indices to uniquely identify each sample. After sequencing, reads must be assigned *in silico* to the sample of origin, a process referred to as demultiplexing. Demultiplexing software typically identifies the sample of origin using a fixed number of mismatches between the read index and a reference index set. This approach may fail or misassign reads when the sequencing quality of the indices is poor.

**Results:** We introduce deML, a maximum likelihood algorithm that demultiplexes Illumina sequences. deML computes the likelihood of an observed index sequence being derived from a specified sample. A quality score which reflects the probability of the assignment being correct is generated for each read. Using these quality scores, even very problematic datasets can be demultiplexed and an error threshold can be set.

**Availability and implementation:** deML is freely available for use under the GPL (http://bioinf.eva.mpg.de/deml/).

**Contact:** gabriel.reno@gmail.com or kelso@eva.mpg.de

**Supplementary information:** Supplementary data are available at *Bioinformatics* online.

## 1 Introduction

While the high-throughput of next generation sequencing is beneficial for many applications, such as high coverage whole-genome sequencing, it may be economically disadvantageous for the sequencing of small numbers of loci. It is possible to sequence large number of samples in a single run by incorporating unique sequence indices for each sample, a process referred to as multiplexing. Current Illumina protocols allow for 1 or 2 index sequences to be used.

The computational process by which reads are assigned to the sample of origin is called demultiplexing. The default demultiplexer provided by Illumina in the CASAVA package allows for 0 or 1 mismatches between the sequenced index and the user-supplied reference indices. Various heuristics have been proposed to assign reads to their sample of origin (Costea *et al*., 2013; Davis *et al*., 2013; Dodt *et al*., 2012; Reid *et al*., 2014).

Although these methods perform well for sequencing reads with high quality, poor demultiplexing remains a common reason for low retrieval or misassignment of sequences from a multiplexed run. Increased error rates—particularly during sequencing of the index—can lead to a higher number of mismatches and hinders assignment to the correct sample. For some applications, high read error rates can be tolerated as long as the reads can be mapped to the reference (e.g. transcriptome quantification).

We introduce deML, a new approach to demultiplexing samples based on likelihood of assignment to a particular sample and provide a freely available, open source C++ implementation. Briefly, we compute the likelihood of a read to originate from each of the original samples, assign reads to the most likely sample of origin and compute the overall confidence in this assignment. We show that by using thresholds on these confidence values, even very problematic datasets can be safely demultiplexed. By simulating increasing error in the indices we show that, especially at high error rates, deML with default quality cutoffs enables the user to demultiplex several fold more sequences than the vendor’s default demultiplexer or other methods based on fixed mismatches. The false discovery rate (FDR) remains below that of other tools based on hamming distance. deML, licensed under the GPL, can run on aligned or unaligned BAM files or FASTQ files.

## 2 Methods

### 2.1 Algorithm

We compute the likelihood of assignment of a read to all potential samples of origin, assign each read to the most likely sample and compute the uncertainty of the assignment.

Let $I=i_{1},i_{2},\ldots ,i_{14}$ be the bases for a specific sample and $R=r_{1},r_{2},\ldots ,r_{14}$ be the two sequenced indices with their respective quality scores $Q=q_{1},q_{2},\ldots ,q_{14}$. Let *m_i_* be a set of dummy variables which are equal to 1 if the corresponding bases between *R* and *I* match, or 0 otherwise. The likelihood of having sequenced the index given that it originates from a given sample, referred to as *Z*_0_, is given by:

(1) $$Z_{0}=-10\cdot {\text{log}}_{10}[\prod_{i=1}^{14}m_{i}\cdot (1-10^{\frac{-q_{i}}{10}})+(1-m_{i})\cdot 10^{\frac{-q_{i}}{10}}]$$

The *Z*_0_ score is computed for each potential match. Finally, the read is assigned to the most likely sample of origin. It can occur that a read is equally likely to belong to more than one sample. To quantify this uncertainty, the *Z*_1_ score models the probability of misassignment. Let *M* be the number of potential samples of origin and let $Z_{0_{1}},Z_{0_{2}},\ldots ,Z_{0_{M}}$ be the likelihood scores for each sample. Let *t* be the sample with the highest likelihood, the misassignment score is given by:

(2) $$Z_{1}=-10\cdot {\text{log}}_{10}[\frac{\sum_{i\in (1..M)\setminus t}10^{\frac{-Z_{0_{i}}}{10}}}{\sum_{j\in (1..M)}10^{\frac{-Z_{0_{j}}}{10}}}]$$

Additional details about the algorithm are found in the Supplementary Methods section.

To evaluate the correctness of the sample assignment based on the indices, we produced double-indexed DNA libraries from amplicons of a 245 bp region of chromosome 7 from 99 human samples and from PhiX DNA fragmented to 350 bp. Double-indexing is increasingly used in applications requiring extremely accurate read assignment (Kircher *et al.*, 2012). The reads were basecalled, demultiplexed using deML and mapped to both the human genome and the PhiX genomes (see Supplementary Methods). The mapping of the forward and reverse reads indicates the sample of origin of the original cluster and was used to measure demultiplexing misassignments rates.

Using simulations, we evaluated the robustness of deML read assignments for datasets at various error rates. Indices with perfect matches to an known sample had sequencing errors were added to them at various rates using an error profile derived from an Illumina MiSeq sequencing run. We computed the number of sequences demultiplexed by deML and by deindexer (https://github.com/ws6/deindexer), which allows users to increase the number of mismatches. We also measured the number of sequences with 0 or 1 mismatches as the standard Illumina demultiplexing approach (CASAVA) assigns sequences using this cutoff (see Supplementary Methods).

## 3 Results

Of the total of 15 245 844 clusters that were detected in our test dataset, 8 070 867 clusters had both forward and reverse reads aligning to the human control region and 4 629 687 to the PhiX. Using the sample assignment provided by deML for the reads mapping to the PhiX, the rate of false assignment was computed as a function of *Z*_0_ and *Z*_1_ scores. As expected, reads with a high likelihood of stemming from the PhiX control (*Z*_0_) group and with a low likelihood of stemming from another sample (*Z*_1_) were enriched for true assignments, whereas misassignments were found at the other end of the distribution. The distribution of the *Z*_0_ and *Z*_1_ scores for true and false positives (TP and FP) are presented in the Supplementary Results.

**Fig. 1.**
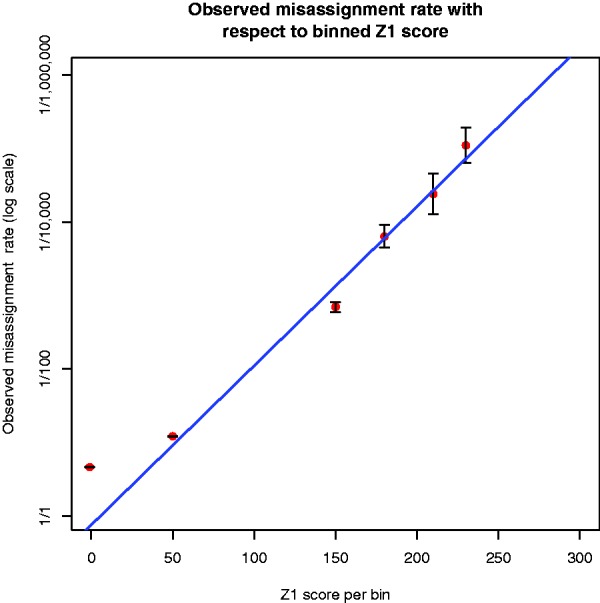
Correlation between the *Z*_1_ score for reads aligned to the PhiX genome and the observed misassignment rate. Error bars were obtained using Wilson score intervals

As *Z*_1_ measures the probability of misassignment given the potential index sequence set on a PHRED scale, the relationship between the misassignment rate on a log scale and the *Z*_1_ score should be linear. For reads where both mates aligned to the PhiX, the misassignment rate was computed by considering any read pair not assigned by deML to the PhiX as a mislabeling. As *Z*_1_ can take many discrete values, the misassignment rate was plotted for multiple *Z*_1_ value bins (see Fig. 1).

deML retrieves more sequences and achieves a lower FDR than currently available approaches (see Table 1 and Supplementary Results).

**Table 1.** Number of sequences demultiplexed by deML and deindexer in terms of TP, FP and FDR for 12 374 149 sequences

Average error	deML			deindexer			CASAVA	
Rate per base	TP	FP	FDR	TP	FP	FDR	0 mm	1 mm
0.002408	12 374 119	1	(0.00%)	12 372 007	0	(0.00%)	11 962 540	405 318
0.101145	11 898 460	205	(0.00%)	9 784 321	146	(0.00%)	2 783 384	4 381 588
0.196708	9 779 898	2761	(0.03%)	5 659 886	1683	(0.03%)	577 456	1 978 848

*Note*: The remaining columns present the number that could be identified using an approach allowing 1 mismatch (such as CASAVA).

## Supplementary Material

Supplementary Data
