# Supplementary material for: deML: robust demultiplexing of Illumina sequences using a likelihood-based approach
Source: Bioinformatics. 2014 Oct 30;31(5):770–2. doi: 10.1093/bioinformatics/btu719 (PMC4341068; doi:10.1093/bioinformatics/btu719)
Supplement: Supplementary Data [file supp_31_5_770__index.html]

deML: robust demultiplexing of Illumina sequences using a likelihood-based approach — deML: robust demultiplexing of Illumina sequences using a likelihood-based approach — Supplementary Data 

# deML: robust demultiplexing of Illumina sequences using a likelihood-based approach

## Supplementary Data

files

**Files in this Data Supplement:**

- Supplementary Data - pdf file
