# Supplementary material for: deML: robust demultiplexing of Illumina sequences using a likelihood-based approach
Source: Bioinformatics. 2014 Oct 30;31(5):770–2. doi: 10.1093/bioinformatics/btu719 (PMC4341068; doi:10.1093/bioinformatics/btu719)
Supplement: Supplementary Data [file supp_btu719_BIOINFO-supp_GR7_MM.pdf]

1 SUPPLEMENTARY METHODS

1.1 Probability of mispairing

The main text describes scores Z_0 and Z_1. Increasingly, sequencing groups use two separate and distinct indices, one on each adapter, a procedure referred to as double indexing (see Kircher *et al.* (2012)). Double indexing offer the possibility to increase the amount of samples that can be pooled on a single run and to increase demultiplexing accuracy. However, a problem that can arise when using a double indexing protocol is incorrect pairing i.e. where the most likely sample of origin for the first index is different from the most likely sample of origin for the second index. In such cases we compute a score that gives the likelihood of incorrect pairing. This score (Z_2) is an approximation of the log odds ratio of the likelihood of mispairing over all possible pairs.

Let $r_{p7} = r_1, r_2, .., r_7$ and $r_{p5} = r_8, r_9, .., r_{14}$ be the two sequenced indices for the p7 and p5 adapters respectively. Let $I7_i$ and $I5_i$ the sequences used for sample i which are considered to be the template. The probability ($p(r_{p7}|I7_i)$ of observing the sequenced data r_{p7} given that $I7_i$ was the template can be computed the same way as the Z_0 score but using only the 7 available nucleotides. To quantify the risk of mispairing, we consider the log odds ratio of the probability of mispairing to the sum of all probabilities for all pairs:

$$Z_2 = \frac{\sum\limits_{i \neq j} p(r_{p7}|I7_i) \cdot p(r_{p5}|I5_j)}{\sum\limits_{i,j} p(r_{p7}|I7_i) \cdot p(r_{p5}|I5_j)} \tag{1}$$

However, the computation above is expensive. A potential way to speed it up is to consider certain terms as being negligible. If the best hit for both P7 and P5 stems from the same sample $\hat{i}$. Let the second best hit be $\hat{j}$ for each index. We can assume that remaining pairs are insignificant compared to the probability of pertaining to these two groups, equation 1 becomes:

$$Z_2 \approx \frac{1}{2} \cdot \frac{p(r_{p7}|I7_{\hat{i}}) \cdot p(r_{p5}|I5_{\hat{j}}) + p(r_{p7}|I7_{\hat{j}}) \cdot p(r_{p5}|I5_{\hat{i}})}{p(r_{p7}|I7_{\hat{i}}) \cdot p(r_{p5}|I5_{\hat{i}})} \tag{2}$$

The scaling factor $\frac{1}{2}$ is due to the use of two terms in the numerator. The log of this expression is expressed as the Z_2 score:

$$Z_2 \approx -0.3 + log_{10}(p(r_{p7}|I7_{\hat{i}}) \cdot p(r_{p5}|I5_{\hat{j}}) + p(r_{p7}|I7_{\hat{j}}) \cdot p(r_{p5}|I5_{\hat{i}})) - log_{10}(p(r_{p7}|I7_{\hat{i}}) \cdot p(r_{p5}|I5_{\hat{i}})) \tag{3}$$

If the best hit for both P7 and P5 does not come from the same sample but rather two different ones, namely $\hat{i}$ and $\hat{j}$, equation 1 can be written as :

$$Z_2 \approx \frac{2}{1} \cdot \frac{p(r_{p7}|I7_{\hat{i}}) \cdot p(r_{p5}|I5_{\hat{j}})}{p(r_{p7}|I7_{\hat{i}}) \cdot p(r_{p5}|I5_{\hat{i}}) + p(r_{p7}|I7_{\hat{j}}) \cdot p(r_{p5}|I5_{\hat{j}})} \tag{4}$$

Again, taking the log yields the Z_2 score:

$$Z_2 \approx 0.3 + log_{10}(p(r_{p7}|I7_{\hat{i}}) \cdot p(r_{p5}|I5_{\hat{j}})) - log_{10}(p(r_{p7}|I7_{\hat{i}}) \cdot p(r_{p5}|I5_{\hat{i}}) + p(r_{p7}|I7_{\hat{j}}) \cdot p(r_{p5}|I5_{\hat{j}})) \tag{5}$$

1.2 Algorithm

For a given observed sequenced index, deML needs to identify all possible index sequences from a user supplied list within a given number of mismatches. To achieve this in a timely fashion, deML builds a prefix tree of the user supplied indices which represent common prefixes as common paths in the tree (see Figure 1). The height of a given node directly indicates the position in the original index string.

An advantage of prefix trees is the ability to search with mismatches using recursive calls in the data structure. The call is launched on the root using the string to be searched and the tolerated number of mismatches. The recursive call is performed on the child nodes where the number of tolerated mismatches is decreased by one when one the letter represented by the current node differs or leaving the mismatch count as is otherwise. The query sequence is shortened by one after each function call. The recursion ends when the number of tolerated mismatches falls below zero or a leaf node is reached.

The overall prefix tree algorithm returns all possible indices within a fixed number of tolerated mismatches for downstream computations. Once all the indices have been identified, the likelihood of pertaining to each sample that has been detected is computed. As mentioned in the main text, upon computing sample assignment quality Z_1, the number of tolerated mismatches can be set to be lesser than the length of the indices as the contribution of the more divergent indices (edit distance exceeding the number of tolerated mismatches) can be generally considered negligible. As the likelihood of pertaining to samples having indices with low edit distance dwarfs the one to samples with more divergent indices, their contribution can often be safely overlooked.

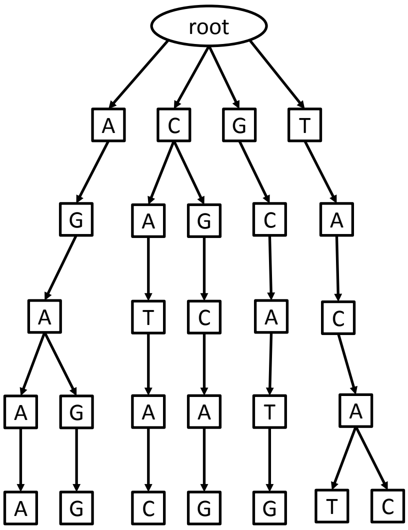

Supplementary Figure 1: A prefix tree for the following sequences : AGAAT,AGAGG,CATAC,CGCAG,GCATG,TACAC,TACAT. The common prefixes become common paths in the tree.

2 SUPPLEMENTARY DATA

2.1 Testing data

A 245 bases long fragment was amplified from human iPS cells digested in QuickExtract DNA Extraction Solution (epibio) using primers GGCTTAAGTCCTGCTGAGA and AGATAAATATAGAATAAAGCTCATGA. Each 25 l PCR reaction contained Phusion HF master mix (NEB) at 1X, each primer at 0.5 M, 0.024 l of template and the rest was water. The mixture was heated to 98C for 30 seconds, followed by 25 cycles of 98C for 10 seconds, 56C for 10 seconds and 72C for 10 seconds. PhiX DNA was fragmented with Covaris S2 with the 500 bases settings (duty cycle 5%; Intensity 3; cycle per burst 200; time 80s) which gave a fragments that had a mod length of 580 bases as judged by a 2100 Bioanalyzer (Agilent). Indexed Illumina libraries were prepared as described by Kircher *et al.* (2012) and the indices used are given in Supplementary Table 1.

deML relies heavily on the base quality scores to compute the likelihood of pertaining to a given sample for a given read. In theory, the base quality scores reflect the probability of error and can therefore be used to accurately compute the probability of observing the bases from the read given a certain sequence template. It is therefore important for quality scores to accurately reflect the probability of error for a given base. We used freeIbis (Renaud *et al.* (2013)) using quality score calibration. While plotting the resulting base quality scores against the observed error rate (see Supplementary Figure 2), the ones predicted by freeIbis show significant improvement in terms of correlation upon comparison with the quality scores predicted by the default basecaller provided by the vendor, Bustard. However, we show (see Supplementary Results Section) that the correlation between our assigned confidence and observed false assignment rates as well as our robustness to error also hold true for Bustard basecalled data. Sequence adapters were removed using cutadapt v1.2.1 and reads shorter than 10 basepairs were removed (Martin (2011)). The reads were demultiplexed using deML[1] according to the list of 100 pairs of index sequences that was used. Reads were aligned to a concatenation of the human genome (1000 Genomes) along with decoy sequences (1000 Genomes decoy sequences version 5) and the PhiX genome sequence (sequence provided by Illumina Corp.) using BWA version 0.5.10 (parameters: “-n 0.01” and no seeding).

[1] github revision:6f503664153b5a2a62678992b05fd722c0a28700

Table 1. Read groups used in this study along with the sequence of the indices and Illumina index numbers.

Name	P7 sequence	P5 sequence	P7 index	P5 index	Name	P7 sequence	P5 sequence	P7 index	P5 index
PCR1	AATTCAA	CATCCGG	341	33	PCR51	CCTAGGT	CGTATAT	303	91
PCR2	CGCGCAG	TCATGGT	342	34	PCR52	GGATCAA	GCTAATC	304	92
PCR3	AAGGTCT	AGAACCG	343	35	PCR53	GCAAGAT	GACTTCT	305	93
PCR4	ACTGGAC	TGGAATA	344	36	PCR54	ATGGAGA	GTACTAT	306	94
PCR5	AGCAGGT	CAGGAGG	345	37	PCR55	CTCGATG	CGAGATC	307	95
PCR6	GTACCGG	AATACCT	346	38	PCR56	GCTCGAA	CGCAGCC	308	96
PCR7	GGTCAAG	CGAATGC	347	39	PCR57	AGTCAGA	CATCCGG	349	33
PCR8	AATGATG	TTCGCAA	348	40	PCR58	AACTAGA	TCATGGT	350	34
PCR9	AGTCAGA	AATTCAA	349	41	PCR59	CTATGGC	AGAACCG	351	35
PCR10	AACTAGA	CGCGCAG	350	42	PCR60	CGACGGT	TGGAATA	352	36
PCR11	CTATGGC	AAGGTCT	351	43	PCR61	AACCAAG	CAGGAGG	353	37
PCR12	CGACGGT	ACTGGAC	352	44	PCR62	CGGCGTA	AATACCT	354	38
PCR13	AACCAAG	AGCAGGT	353	45	PCR63	GCAGTCC	CGAATGC	355	39
PCR14	CGGCGTA	GTACCGG	354	46	PCR64	CTCGCGC	TTCGCAA	356	40
PCR15	GCAGTCC	GGTCAAG	355	47	PCR65	CTGCGAC	AATTCAA	357	41
PCR16	CTCGCGC	AATGATG	356	48	PCR66	ACGTATG	CGCGCAG	358	42
PCR17	CTGCGAC	AGTCAGA	357	49	PCR67	ATACTGA	AAGGTCT	359	43
PCR18	ACGTATG	AACTAGA	358	50	PCR68	CAGGAGG	CCGATTG	337	29
PCR19	ATACTGA	CTATGGC	359	51	PCR69	AATACCT	ATGCCGC	338	30
PCR20	TACTTAG	CGACGGT	360	52	PCR70	CGAATGC	CAGTACT	339	31
PCR21	AAGCTAA	AACCAAG	361	53	PCR71	TTCGCAA	AATAGTA	340	32
PCR22	GACGGCG	CGGCGTA	362	54	PCR72	ACCAACT	TCGCAGG	309	1
PCR23	AGAAGAC	GCAGTCC	363	55	PCR73	CCGGTAC	CTCTGCA	310	2
PCR24	GTCCGGC	CTCGCGC	364	56	PCR74	AACTCCG	CCTAGGT	311	3
PCR25	TTCAACC	TCAGCTT	373	65	PCR75	TTGAAGT	GGATCAA	312	4
PCR26	TTAACTC	AGAGCGC	374	66	PCR76	ACTATCA	GCAAGAT	313	5
PCR27	TAGTCTA	GCCTACG	375	67	PCR77	TTGGATC	ATGGAGA	314	6
PCR28	TGCATGA	TAATCAT	376	68	PCR78	CGACCTG	CTCGATG	315	7
PCR29	AATAAGC	AACCTGC	377	69	PCR79	TAATGCG	GCTCGAA	316	8
PCR30	AGCCTTG	GACGATT	378	70	PCR80	AGGTACC	ACCAACT	317	9
PCR31	CCAACCT	TAGGCCG	379	71	PCR81	TGCGTCC	CCGGTAC	318	10
PCR32	GCAGAAG	GGCATAG	380	72	PCR82	GAATCTC	AACTCCG	319	11
PCR33	AGAATTA	TTCAACC	381	73	PCR83	CATGCTC	TTGAAGT	320	12
PCR34	CAGCATC	TTAACTC	382	74	PCR84	ACGCAAC	ACTATCA	321	13
PCR35	TTCTAGG	TAGTCTA	383	75	PCR85	GCATTGG	TTGGATC	322	14
PCR36	CCTCTAG	TGCATGA	384	76	PCR86	GATCTCG	CGACCTG	323	15
PCR37	CCGGATA	AATAAGC	385	77	PCR87	CAATATG	TAATGCG	324	16
PCR38	GCCGCCT	AGCCTTG	386	78	PCR88	TGACGTC	AGGTACC	325	17
PCR39	AACGACC	CCAACCT	387	79	PCR89	GATGCCA	TGCGTCC	326	18
PCR40	CCAGCGG	GCAGAAG	388	80	PCR90	CAATTAC	GAATCTC	327	19
PCR41	TAGTTCC	AGAATTA	389	81	PCR91	AGATAGG	CATGCTC	328	20
PCR42	TGGCAAT	CAGCATC	390	82	PCR92	CCGATTG	ACGCAAC	329	21
PCR43	CGTATAT	TTCTAGG	391	83	PCR93	ATGCCGC	GCATTGG	330	22
PCR44	GCTAATC	CCTCTAG	392	84	PCR94	CAGTACT	GATCTCG	331	23
PCR45	GACTTCT	CCGGATA	393	85	PCR95	AATAGTA	CAATATG	332	24
PCR46	GTACTAT	GCCGCCT	394	86	PCR96	CATCCGG	TGACGTC	333	25
PCR47	CGAGATC	AACGACC	395	87	PCR97	TCATGGT	GATGCCA	334	26
PCR48	CGCAGCC	CCAGCGG	396	88	PCR98	AGAACCG	CAATTAC	335	27
PCR49	TCGCAGG	TAGTTCC	301	89	PCR99	TGGAATA	AGATAGG	336	28
PCR50	CTCTGCA	TGGCAAT	302	90	PhiX	GACGATT	GACGGCG	370	62

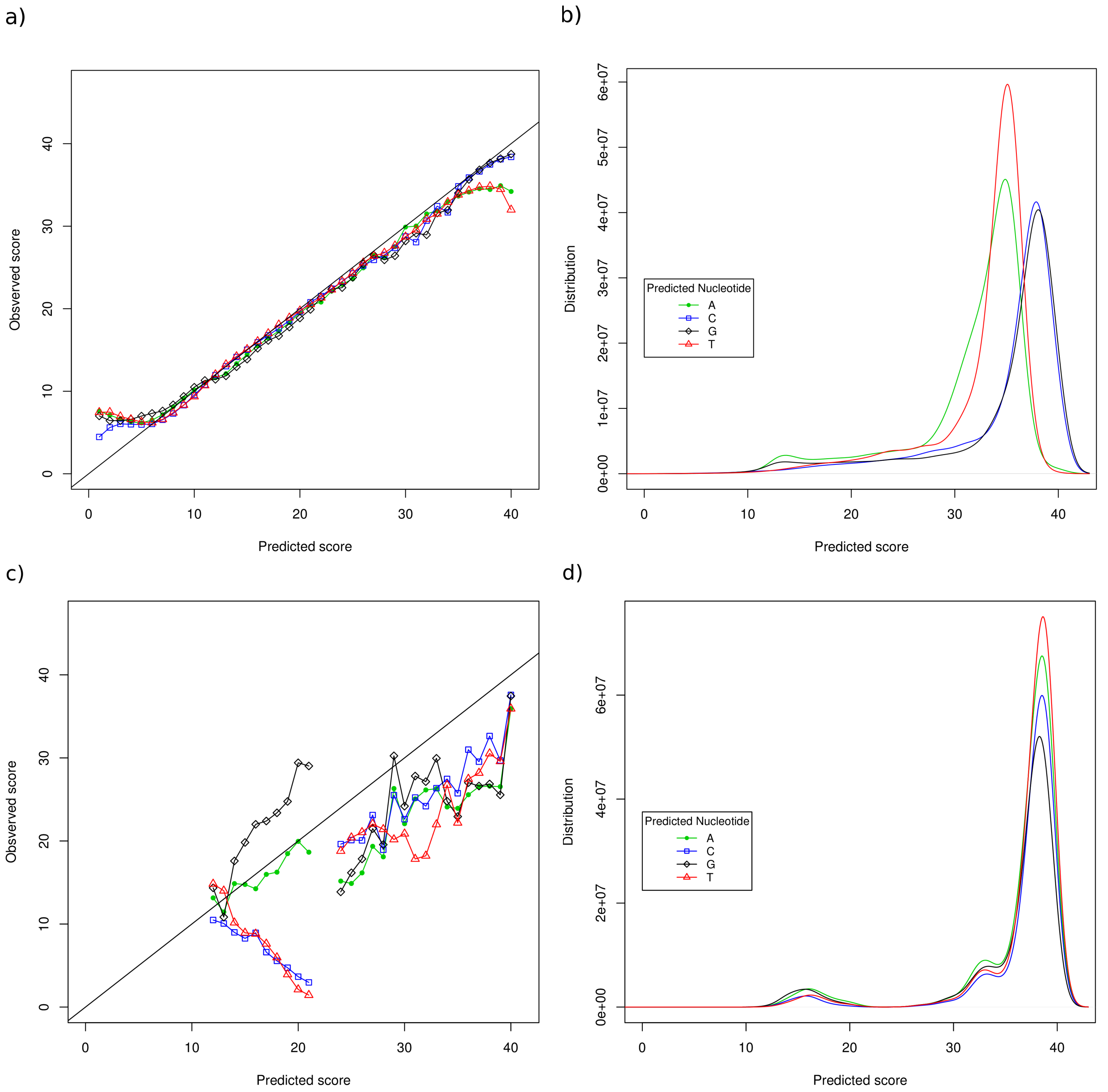

Supplementary Figure 2: Quality scores for the MiSeq run described in this study. **(a)** The predicted versus observed quality scores for the sequenced bases basecalled using freeIbis. Ideally, the quality scores should follow the diagonal. **(b)** The distribution of the quality scores using a standard density plot for each nucleotide. **(c)** The predicted versus observed quality scores for the sequenced bases from the default basecaller. **(d)** The distribution of the quality scores provided by the default Illumina basecaller.

Table 2. A tally for every possible combination of the forward and reverse read placement for the Illumina MiSeq presented in this study.

position of first mate	position of second mate	number
PCR product	PCR product	8,070,867
PCR product	phix	234
PCR product	outside targets	56
PCR product	unmapped	545,285
phix	PCR product	179
phix	phix	4,629,687
phix	outside targets	14
phix	unmapped	211,156
outside targets	PCR product	11
outside targets	phix	1
outside targets	outside target	10,084
outside targets	unmapped	24,496
unmapped	PCR product	241,960
unmapped	phix	66,132
unmapped	outside targets	22,470
unmapped	unmapped	1,368,465

3 SUPPLEMENTARY RESULTS

3.1 Mapping statistics

As the two potential templates of the sequencing run were human genome PCR product and PhiX controls, we expect most reads to map to either one of those regions. We analyzed each pair of reads and considered that a read can fall within 4 categories: PCR region, PhiX, mapped to a region outside our targets and unmapped. The tally (see Table 2) shows that most pairs are either both PCR product or both PhiX.

3.2 Discordant pairs

A small number of read pairs exhibited unexpected mapping patterns. For 179 clusters, the first mate mapped to PhiX and second one mapped to the PCR region. A total of 234 clusters exhibit the converse pattern where the first mate mapped to the PCR region but the second mapped to the PhiX genome. For those 413 (179+234) clusters, we sought to test the possibility that they might have been generated by shifting clusters and therefore have a high probability of error. For a read of length L and where q_l is the PHRED quality score for the base at position l, the expected number of mismatches per base to the reference is computed using the following expression:

$$\frac{\sum_{l=1}^{L} 10^{\frac{-q_l}{10}}}{L} \quad (6)$$

The expression above is the average number of mismatches for any given base and can be multiplied by a sequence length to know how many mismatches are expected over the entire molecule. The expected number of mismatches for this subset was calculated to be 0.0301 or essentially 3.01 mismatches per 100 bases. To assess whether this number is higher in a statistically significant way, 10,000 subsets of 413 cluster were selected at random from the initial BAM file. The distribution for the expected number of mismatches for those randomized subsets were plotted (see Figure 3) against the same number of these discordant pairs. The expected number of mismatches is higher than any of the random subsets ($p<0.0001$).

3.3 Distribution of predictive scores

For reads aligning to the PhiX genome, we consider reads demultiplexed as control to be unequivocally true assignments and human PCR samples to be false assignments. Out of the 8,286,275 unique reads mapping with high mapping quality (MAPQ>=30) to the PhiX genome, 42,089 (0.51%) reads were demultiplexed as one of the human PCR samples. To show that the scores produced by deML can minimize these false assignments, the correlation between these scores and true and false assignments was evaluated. This computation was done on the set of false assignments and a subset of equal size of true assignments.

For the Z_0 score (see Figure 4a), the majority of true assignments (green) have a high probability of pertaining to the sample to which they were assigned. False assignments (red) have on average a much lower probability of pertaining to the sample of origin as shown by the higher Z_0 score. The density of Z_1 score, the probability of pertaining to another sample than the most likely one, was also plotted (see Figure 4b). True assignments (green) have a lower probability of misassignment compared to actual misassignments (red).

Table 3. Predictive value of the Z_0, Z_1 and both scores used in conjunction to classify correct assignments from mis-assignments using the data presented in Figure 1 in the main text.

predictor	misclassification out of 84,178
Z_0	1,751
Z_1	1,628
Z_0 and Z_1	1,606

To measure the joint distribution of the Z_0 and Z_1 for correct and false assignments, the distribution of both scores were projected for the same reads described in the paragraph above. The distribution of Z_0 and Z_1 scores of false assignments and a subset of equal size of true assignments was plotted (see Figure 5). The color of the individual dots depended on the density of other dots in the vicinity. As expected, reads with a high likelihood of stemming from the PhiX control (Z_0) group and with a low likelihood of stemming from another sample (Z_1) were enriched for true assignments whereas misassignments were found at the other end of the distribution.

3.4 Predictive power of combined scores

To evaluate whether having both Z_0 and Z_1 scores has better predictive power than solely using a single one, a logistic regression was performed using each score individually and both at once. Using the PhiX data presented in the main text in Figure 1, positive and negative assignments were used as labels and the Z_0 and Z_1 scores were used as potentially predictive values. The classification was performed using a logistic regression using the glm() function in R version 3.0.1. For all 3 set (Z_0, Z_1 and Z_0, Z_1 combined), the number of misclassifications were computed. The lowest number of misclassifications were obtained using both scores in conjunction.

3.5 Robustness to sequencing errors

To evaluate the robustness of the demultiplexing to increased error rates, reads with perfect matches to index sequences from a known sample were taken from the original set and mismatches were added using an Illumina error profile. This profile contains sequencer-specific nucleotide substitutions along with quality scores for those. As CASAVA returns only sequences with a maximum of 1 mismatch, the number of sequences with 0 mismatches, 1 mismatch and 2 or more mismatches to the original indices are presented (see Table 4). We ran deML using the default cutoffs ($Z_0 > 80$ and $Z_1 > 20$). For comparison, we ran deindexer (https://github.com/ws6/deindexer [2], which detects matches to known sample indices using hashes to detect collisions and avoid false assignments. For deindexer, at most 5 mismatches were tolerated as two random sequences of 7 basepairs will exhibit on average 5 mismatches ($7 \cdot \frac{3}{4} = 5.25$).

At higher error rates, the number of demultiplexed sequences that could be retrieved by CASAVA substantially as sequences with 1 mismatch or less become a small fraction of the total. However, deML shows greater robustness to increased error rates while keeping a mis-assignment rate under 0.5% even at very high error rates for sequences meeting the default thresholds. The other software, deindexer, performs well at low error rates but does not scale well to high error rates (see Table 5). At the highest simulated error rate, deML demultiplexes 3.1 times more sequences while maintaining a lower false discovery rate (0.44% for deML and 0.56% for deindexer).

3.6 Speed versus sensitivity

As the maximum number of allowed mismatches can be specified from the command line, the sensitivity and runtime of the program were evaluated as a function of the number of mismatches (see Table 6). Tolerating more mismatches leads to slower execution speeds but allows greater sensitivity and a more accurate calculation of the Z_1 score.

At 3 mismatches, deML was able to detect at least one sample of origin for each sequence. It is worth noting that the quality of this sequencing run used in this study was excellent and allowing additional mismatches was not needed to find at least one sample of origin for each sequence.

3.7 Background error rate

As mentioned in the discussion, if we consider only clusters with a high probability of pertaining to their respective read group ($Z_0 = 0$), where both pairs map to the PhiX, the overwhelming majority were demultiplexed as Phix. However, there were 9 clusters (18 sequences in total) which were assigned to the human PCR region samples. In theory, such sequences with indices matching to samples pertaining to PCR regions with perfect matches yet where the forward and reverse read match to the PhiX control should not exist. To investigate whether mixed clusters could have produced such sequences, the expected number of mismatches per base for those 18 sequences were computed.

[2] revision: 69999c0c049919f1caa3ed35a69c7592203f7b18

The same quantity was computed for 10,000 independently sampled subsets of 18 sequences for the entire dataset. A comparison reveals that those sequences do not have an expected number of mismatches per base above those of the background (see Figure 6) thus making the mixed cluster hypothesis unlikely.

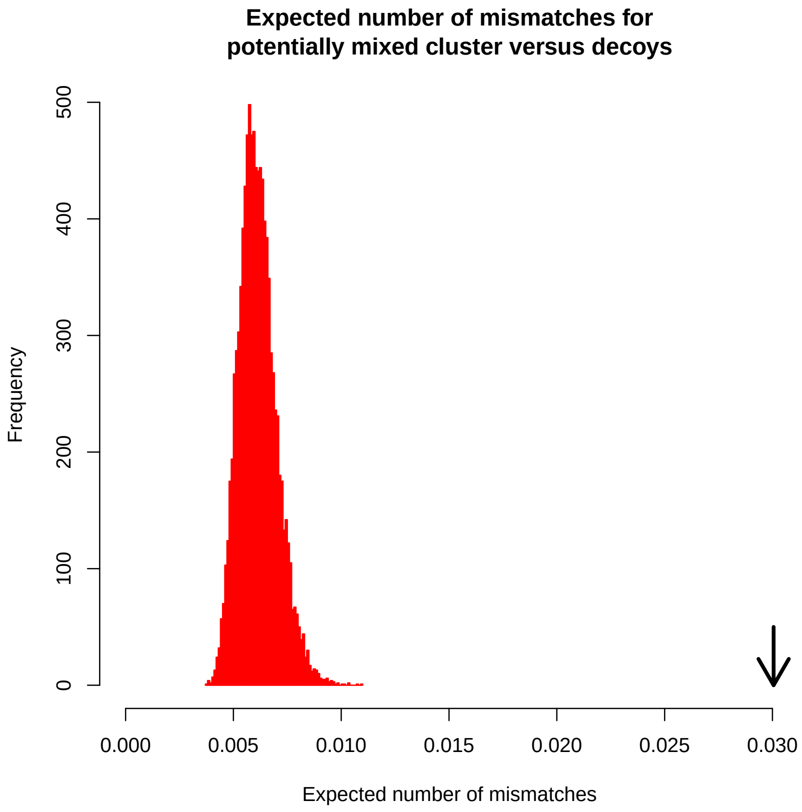

Supplementary Figure 3: The expected number of mismatches per base for the 413 discordant pairs (e.g. one mate mapping to the PCR human target, the other mapping to the PhiX genome) is represented as an black arrow. The distribution of the expected number of mismatches per base for 10,000 subsets of 413 pairs were taken at random is represented in red.

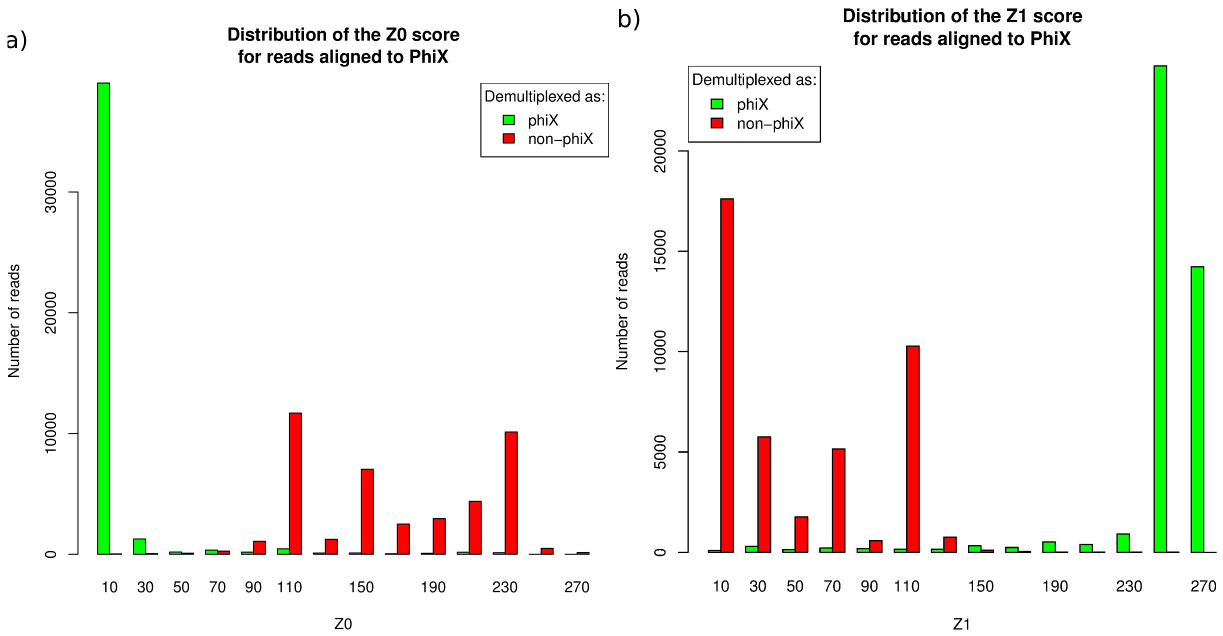

Supplementary Figure 4: a) Distribution of the Z_0 score for reads aligning for the PhiX genome for reads either demultiplexed as control (green) or as human samples (red) b) Distribution for the same reads but for the Z_1 score.

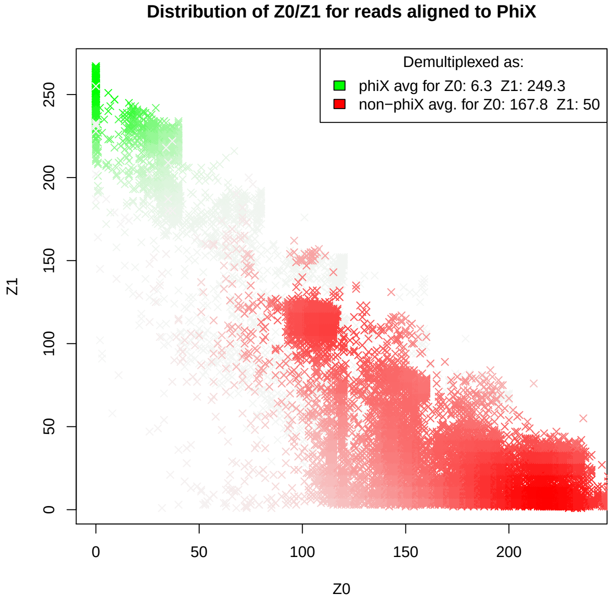

Supplementary Figure 5: Distribution of true assignments and false assignments and their respective Z_0 and Z_1 scores for reads aligned to the PhiX depending on whether they were demultiplexed as a human sample (red) or PhiX (green). Reads demultiplexed as being part of the human PCR samples can be considered to be false assignments. A subset using an equal amount of true assignments was used to compare the Z_0 and Z_1 scores. The intensity of the color indicates the density of the data points for the given category. Data points with a dearth of other points from the same category in the vicinity are dyed as gray.

Table 4. Tally of the amount of sequences demultiplexed by deML at various levels of simulated error rates. The number of sequences that could be demultiplexed by an approach that used a cutoff of 1 mismatch like CASAVA is also presented.

avg. error rate per base	correctly assigned QC passed	correctly assigned QC failed	wrongly assigned QC passed	wrongly assigned QC failed	0 mismatches	1 mismatch	2 or more mismatches	error rate for QC passed
0.002408	12,374,119	29	1	0	11,962,540	405,318	6,291	0.00%
0.010169	12,373,301	847	0	1	10,725,994	1,540,905	107,250	0.00%
0.020160	12,368,861	5,277	2	9	9,305,305	2,679,637	389,207	0.00%
0.029793	12,358,306	15,809	3	31	8,105,076	3,481,942	787,131	0.00%
0.039433	12,339,811	34,251	9	78	7,048,970	4,047,523	1,277,656	0.00%
0.048776	12,311,489	62,491	12	157	6,146,987	4,410,820	1,816,342	0.00%
0.057784	12,274,074	99,708	29	338	5,379,913	4,618,279	2,375,957	0.00%
0.066878	12,221,546	151,926	42	635	4,697,957	4,713,000	2,963,192	0.00%
0.075641	12,160,346	212,680	64	1,059	4,119,163	4,712,652	3,542,334	0.00%
0.084253	12,085,534	286,819	89	1,707	3,613,460	4,648,446	4,112,243	0.00%
0.092736	11,998,912	372,511	151	2,575	3,169,831	4,535,227	4,669,091	0.00%
0.101145	11,898,460	471,721	205	3,763	2,783,384	4,381,588	5,209,177	0.00%
0.109136	11,789,483	579,097	260	5,309	2,456,736	4,212,362	5,705,051	0.00%
0.117206	11,664,964	701,423	368	7,394	2,163,380	4,017,595	6,193,174	0.00%
0.125214	11,528,595	835,066	420	10,068	1,903,980	3,813,182	6,656,987	0.00%
0.132844	11,388,127	972,122	534	13,366	1,684,998	3,609,607	7,079,544	0.00%
0.140526	11,234,310	1,122,111	698	17,030	1,486,412	3,399,036	7,488,701	0.01%
0.147897	11,076,751	1,274,925	868	21,605	1,317,939	3,198,341	7,857,869	0.01%
0.155148	10,909,794	1,435,683	1,022	27,650	1,169,888	3,005,939	8,198,322	0.01%
0.162508	10,731,576	1,607,100	1,288	34,185	1,034,347	2,807,681	8,532,121	0.01%
0.169414	10,555,336	1,775,939	1,516	41,358	921,690	2,631,870	8,820,589	0.01%
0.176525	10,362,080	1,959,683	1,757	50,629	815,864	2,452,554	9,105,731	0.02%
0.183351	10,171,497	2,139,740	2,061	60,851	727,790	2,286,576	9,359,783	0.02%
0.190047	9,979,106	2,320,527	2,437	72,079	648,330	2,128,565	9,597,254	0.02%
0.196708	9,779,898	2,506,808	2,761	84,682	577,456	1,978,848	9,817,845	0.03%
0.203133	9,581,645	2,690,309	3,087	99,108	516,142	1,837,545	10,020,462	0.03%
0.209702	9,376,786	2,878,642	3,657	115,064	460,367	1,705,867	10,207,915	0.04%
0.215989	9,170,620	3,067,416	3,966	132,147	410,283	1,583,551	10,380,315	0.04%
0.222165	8,967,960	3,249,621	4,513	152,055	368,415	1,469,445	10,536,289	0.05%
0.228286	8,764,114	3,432,621	4,981	172,433	329,986	1,363,794	10,680,369	0.06%
0.234293	8,563,485	3,610,821	5,398	194,445	294,948	1,261,862	10,817,339	0.06%
0.240130	8,361,952	3,788,095	6,069	218,033	265,964	1,172,482	10,935,703	0.07%
0.245970	8,161,182	3,961,789	6,572	244,606	238,975	1,087,117	11,048,057	0.08%
0.251751	7,960,228	4,134,271	7,191	272,459	214,151	1,005,856	11,154,142	0.09%
0.257444	7,757,196	4,307,519	8,025	301,409	191,882	932,307	11,249,960	0.10%
0.263088	7,559,422	4,472,017	8,819	333,891	172,448	863,078	11,338,623	0.12%
0.268502	7,367,339	4,631,315	9,260	366,235	155,434	799,425	11,419,290	0.13%
0.273824	7,179,504	4,782,441	10,071	402,133	140,083	742,376	11,491,690	0.14%
0.279133	6,990,316	4,934,765	10,947	438,121	126,579	687,184	11,560,386	0.16%
0.284395	6,809,979	5,074,764	11,593	477,813	114,574	637,054	11,622,521	0.17%
0.289590	6,621,120	5,222,775	12,424	517,830	103,693	590,018	11,680,438	0.19%
0.294594	6,445,396	5,355,157	13,339	560,257	93,404	546,745	11,734,000	0.21%
0.299673	6,266,929	5,488,381	14,353	604,486	84,181	507,277	11,782,691	0.23%
0.304587	6,096,095	5,611,609	15,211	651,234	76,847	470,773	11,826,529	0.25%
0.309421	5,928,675	5,731,211	16,346	697,917	69,722	435,871	11,868,556	0.27%
0.314206	5,766,160	5,842,967	17,101	747,921	62,569	405,096	11,906,484	0.30%
0.318938	5,606,108	5,949,529	18,190	800,322	57,340	375,096	11,941,713	0.32%
0.323720	5,447,198	6,053,435	19,140	854,376	51,726	347,396	11,975,027	0.35%
0.328343	5,288,741	6,156,930	20,350	908,128	47,175	322,750	12,004,224	0.38%
0.332825	5,143,582	6,244,076	21,403	965,088	42,879	300,473	12,030,797	0.41%
0.337214	4,997,095	6,334,509	22,246	1,020,299	38,762	278,974	12,056,413	0.44%

Table 5. Demultiplexing accuracy for deindexer using 5 mismatches on the same dataset as Supplementary Table 4. The total number of reads assigned to a sample as well as the percentage out of those that were correctly assigned to their sample of origin is presented.

avg. error rate per base	total assigned	total assigned (percentage)	correctly assigned	correctly assigned (percentage)	wrongly assigned	wrongly assigned (percentage)
0.002408	12,372,007	99.98%	12,372,007	100.00%	0	0.00%
0.010169	12,339,174	99.72%	12,339,174	100.00%	0	0.00%
0.020160	12,241,052	98.92%	12,241,050	100.00%	2	0.00%
0.029793	12,090,199	97.71%	12,090,193	100.00%	6	0.00%
0.039433	11,890,368	96.09%	11,890,358	100.00%	10	0.00%
0.048776	11,655,768	94.19%	11,655,757	100.00%	11	0.00%
0.057784	11,392,014	92.06%	11,391,992	100.00%	22	0.00%
0.066878	11,097,491	89.68%	11,097,456	100.00%	35	0.00%
0.075641	10,792,888	87.22%	10,792,839	100.00%	49	0.00%
0.084253	10,468,656	84.60%	10,468,574	100.00%	82	0.00%
0.092736	10,133,799	81.89%	10,133,673	100.00%	126	0.00%
0.101145	9,784,467	79.07%	9,784,321	100.00%	146	0.00%
0.109136	9,448,900	76.36%	9,448,669	100.00%	231	0.00%
0.117206	9,100,837	73.55%	9,100,558	100.00%	279	0.00%
0.125214	8,750,603	70.72%	8,750,276	100.00%	327	0.00%
0.132844	8,412,618	67.99%	8,412,197	99.99%	421	0.01%
0.140526	8,069,154	65.21%	8,068,666	99.99%	488	0.01%
0.147897	7,742,975	62.57%	7,742,383	99.99%	592	0.01%
0.155148	7,425,134	60.01%	7,424,412	99.99%	722	0.01%
0.162508	7,102,569	57.40%	7,101,706	99.99%	863	0.01%
0.169414	6,803,325	54.98%	6,802,344	99.99%	981	0.01%
0.176525	6,495,529	52.49%	6,494,413	99.98%	1,116	0.02%
0.183351	6,208,271	50.17%	6,206,979	99.98%	1,292	0.02%
0.190047	5,933,211	47.95%	5,931,724	99.97%	1,487	0.03%
0.196708	5,661,569	45.75%	5,659,886	99.97%	1,683	0.03%
0.203133	5,403,335	43.67%	5,401,507	99.97%	1,828	0.03%
0.209702	5,148,329	41.61%	5,146,190	99.96%	2,139	0.04%
0.215989	4,910,033	39.68%	4,907,770	99.95%	2,263	0.05%
0.222165	4,679,976	37.82%	4,677,388	99.94%	2,588	0.06%
0.228286	4,458,765	36.03%	4,456,066	99.94%	2,699	0.06%
0.234293	4,249,556	34.34%	4,246,580	99.93%	2,976	0.07%
0.240130	4,052,215	32.75%	4,048,996	99.92%	3,219	0.08%
0.245970	3,859,842	31.19%	3,856,418	99.91%	3,424	0.09%
0.251751	3,671,336	29.67%	3,667,652	99.90%	3,684	0.10%
0.257444	3,497,703	28.27%	3,493,704	99.89%	3,999	0.11%
0.263088	3,327,230	26.89%	3,322,929	99.87%	4,301	0.13%
0.268502	3,167,027	25.59%	3,162,333	99.85%	4,694	0.15%
0.273824	3,019,710	24.40%	3,014,767	99.84%	4,943	0.16%
0.279133	2,872,426	23.21%	2,867,284	99.82%	5,142	0.18%
0.284395	2,736,192	22.11%	2,730,853	99.80%	5,339	0.20%
0.289590	2,603,918	21.04%	2,598,208	99.78%	5,710	0.22%
0.294594	2,480,293	20.04%	2,474,152	99.75%	6,141	0.25%
0.299673	2,361,410	19.08%	2,355,129	99.73%	6,281	0.27%
0.304587	2,244,057	18.14%	2,237,436	99.70%	6,621	0.30%
0.309421	2,138,234	17.28%	2,131,198	99.67%	7,036	0.33%
0.314206	2,037,391	16.46%	2,030,118	99.64%	7,273	0.36%
0.318938	1,938,339	15.66%	1,930,704	99.61%	7,635	0.39%
0.323720	1,845,527	14.91%	1,837,569	99.57%	7,958	0.43%
0.328343	1,757,207	14.20%	1,748,765	99.52%	8,442	0.48%
0.332825	1,673,441	13.52%	1,664,830	99.49%	8,611	0.51%
0.337214	1,598,108	12.91%	1,589,200	99.44%	8,908	0.56%

Table 6. The runtime and percentage of sequences that remained unassigned to any sample as a function of allowed mismatches.

allowed mismatches	runtime (real)	Percentage unassigned
0	11m15s	6.24%
1	11m16s	1.65%
2	11m55s	0.04%
3	14m38s	0.00%
4	17m58s	0.00%
5	21m44s	0.00%
6	23m41s	0.00%
7	23m34s	0.00%

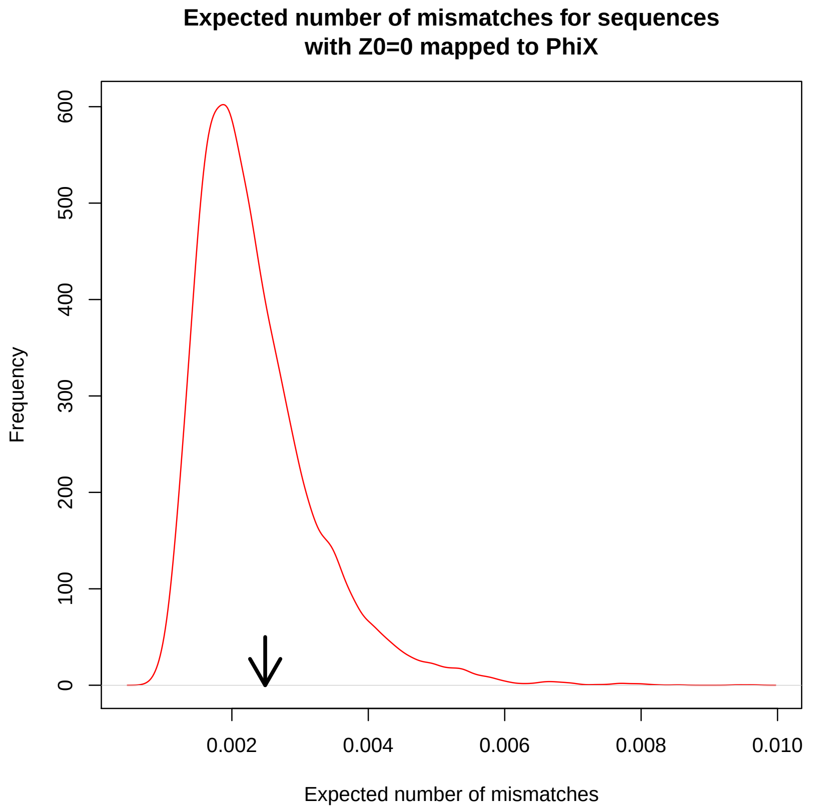

Supplementary Figure 6: The distribution of the expected number of mismatches per base for 10,000 sets of 18 randomly chosen sequences mapping to the PhiX genome with $Z_0 = 0$ (red line) versus the ones not demultiplexed as PhiX but as human PCR region (black arrow).

3.8 Demultiplexing with default quality scores

We show in the Supplementary Data section that, for the MiSeq run used in this study, predicted quality scores produced by Bustard (the default Illumina basecaller) do not have a perfect correlation to their observed ones. Some groups rectify this discrepancy after basecalling using the Genome Analysis Toolkit (GATK) however, this is not feasible for index sequences (McKenna *et al.* (2010)). As deML relies on quality scores, we verified whether the algorithm would work equally well for sequence data produced by the default Illumina basecaller. More precisely, the correlation between the false assignment rate and the Z_0 and the Z_1 scores was evaluated. We demultiplexed the same data but instead of the freeIbis basecalls, the default Illumina basecalls were used. The distribution of the false assignments versus true ones were plotted (see Figure 7). Furthermore, the correlation between the mis-assignment rate and the Z_1 score was also measured (see Figure 8). In both cases, the correlation between both scores and the false assignment rates holds. This is a likely consequence of the fact that quality scores produced by Bustard, albeit not having a perfect correlation to their observed error rates, offer a reasonable approximation for the major part of them (quality scores between 30 and 40). Similarly to freeIbis, the quality scores at the lower end of the distribution (less than 20 on the PHRED scale) do not seem to correspond to their observed error rate. As a consequence, the first data point in Figure 8 does not seem to follow well the predicted linear relationship.

We also evaluated whether deML would provide the same robustness for the data basecalled with Bustard. In an approach identical to the one used for freeIbis basecalled dataset, mismatches in the indices were added at various rates. The substitutions and quality scores for the mismatching bases were added using a Bustard error profile obtained from control sequences aligned to the PhiX. The number of sequences that could be demultiplexed by deML greatly exceeds the retrievable number of sequences using the default strategy of allowing 1 mismatch, especially at high error rates (see Figure 9). Similarly to results obtained on the data basecalled using freeIbis presented in Table 4 in the Supplementary Results, at the highest error rate, this set also had a low amount of false assignments (8,469 sequences) out of those that passed our default quality thresholds (2,605,363 sequences) for a maximal observed false assignment rate of 0.33%.

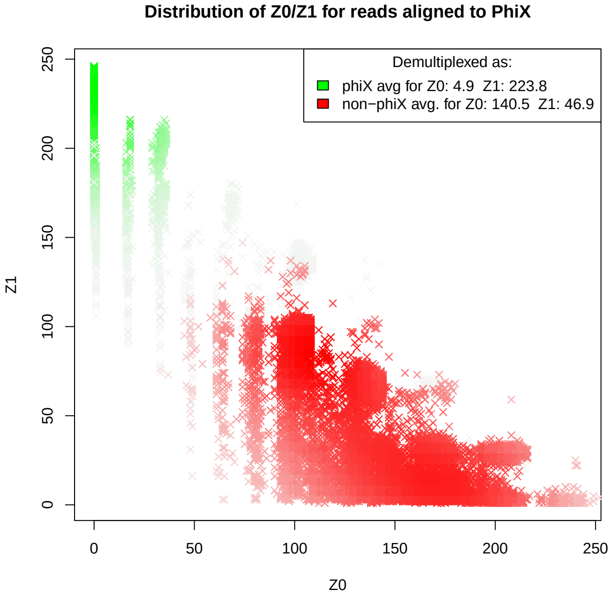

Supplementary Figure 7: Distribution of true assignments (green) and false assignments (red) to the PhiX genome over their respective Z_0 and Z_1 score for reads from the Bustard basecaller. Like figure 5, the intensity of the color indicates the density of the data points for the given category.

REFERENCES

Kircher, M., Sawyer, S., and Meyer, M. (2012). Double indexing overcomes inaccuracies in multiplex sequencing on the illumina platform. *Nucleic acids research*, **40**(1), e3–e3.

Martin, M. (2011). Cutadapt removes adapter sequences from high-throughput sequencing reads. *EMBnet. journal*, **17**(1), pp–10.

McKenna, A., Hanna, M., Banks, E., Sivachenko, A., Cibulskis, K., Kernytsky, A., Garimella, K., Altshuler, D., Gabriel, S., Daly, M., *et al.* (2010). The genome analysis toolkit: A mapreduce framework for analyzing next-generation DNA sequencing data. *Genome research*, **20**(9), 1297–1303.

Renaud, G., Kircher, M., Stenzel, U., and Kelso, J. (2013). freeibis: an efficient basecaller with calibrated quality scores for illumina sequencers. *Bioinformatics*, **29**(9), 1208–1209.

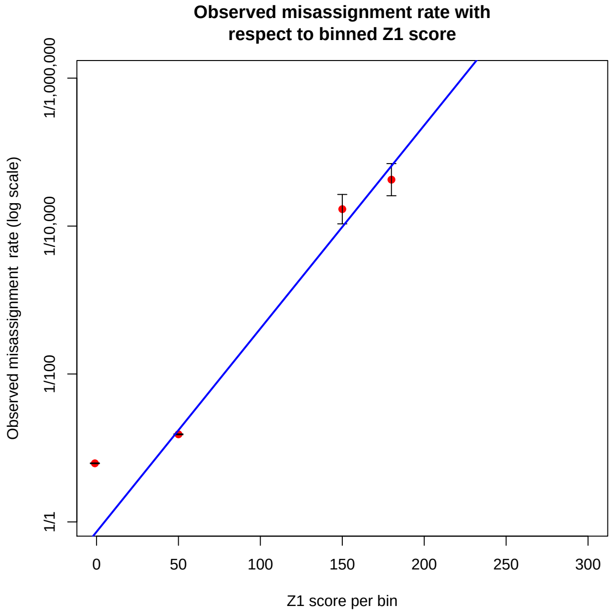

Supplementary Figure 8: Correlation between the Z_1 score for reads aligned to the PhiX genome and the observed mis-assignment rate on a log scale for the Bustard basecalled reads. Like the figure in the main document, the line is a linear regression using on all but the first data points. The size of the bins are the same as the ones used for the main figure except that no false assignments were seen for this dataset for a Z_1 score above 200 hence no datapoint was reported. Error bars were obtained using Wilson score intervals.

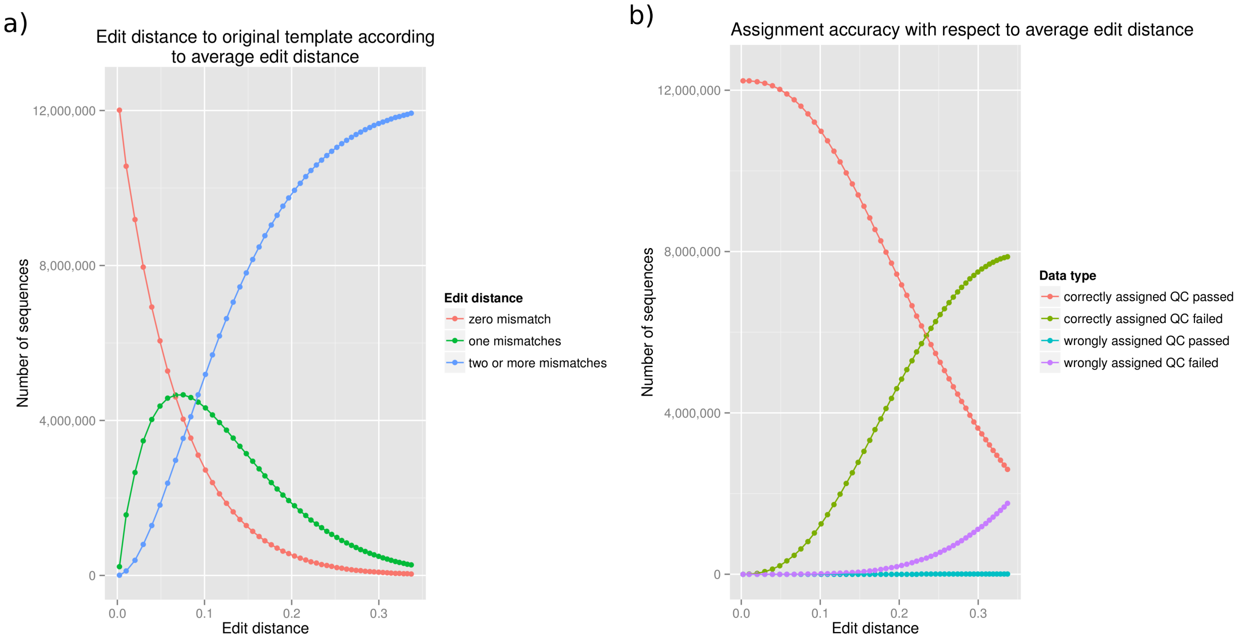

Supplementary Figure 9: a) The edit distance of the simulated indices to the original index sequence as a function of the simulated edit distance to the original indices for Bustard basecalled data. This graph indicates the limits of heuristics using fixed-mismatches like CASAVA. b) For the same dataset, the number of sequences correctly assigned to the original sample for both the ones that passed quality threshold and those that did not. The number of incorrect assignments are also reported for both categories.
